# AKT1 induces Nanog promoter in a SUMOylation-dependent manner in different pluripotent contexts

**DOI:** 10.1186/s13104-023-06598-3

**Published:** 2023-11-03

**Authors:** Marcos Gabriel Francia, Paula Verneri, Camila Oses, Camila Vazquez Echegaray, Mora Reneé Garcia, Ayelen Toro, Valeria Levi, Alejandra Sonia Guberman

**Affiliations:** 1 Instituto de Química Biológica de la Facultad de Ciencias Exactas y Naturales (IQUIBICEN), Departamento de Química Biológica, Facultad de Ciencias Exactas y Naturales, CONICET, Universidad de Buenos Aires, Buenos Aires, Argentina; 2 Departamento de Fisiología y Biología Molecular y Celular, Facultad de Ciencias Exactas y Naturales, Universidad de Buenos Aires, Buenos Aires, Argentina; 3 Present Address: Lund Stem Cell Center, Department of Molecular Medicine and Gene Therapy, Lund University, Lund, Sweden; 4 Laboratorio de Regulación Génica en Células Madre (CONICET-UBA), Intendente Guiraldes 2160 Pab. 2, 4to Piso, QB-71, C1428EGA Buenos Aires, Argentina

**Keywords:** Embryonic stem cells, Induced pluripotent stem cells, AKT1 SUMO conjugation, E17K AKT1 mutant, UBC9(C93S), MEF, U-2 OS

## Abstract

AKT/PKB is a kinase crucial for pluripotency maintenance in pluripotent stem cells. Multiple post-translational modifications modulate its activity. We have previously demonstrated that AKT1 induces the expression of the pluripotency transcription factor Nanog in a SUMOylation-dependent manner in mouse embryonic stem cells. Here, we studied different cellular contexts and main candidates that could mediate this induction. Our results strongly suggest the pluripotency transcription factors OCT4 and SOX2 are not essential mediators. Additionally, we concluded that this induction takes place in different pluripotent contexts but not in terminally differentiated cells. Finally, the cross-matching analysis of ESCs, iPSCs and MEFs transcriptomes and AKT1 phosphorylation targets provided new clues about possible factors that could be involved in the SUMOylation-dependent Nanog induction by AKT.

**Supplementary Information:**

The online version contains supplementary material available at 10.1186/s13104-023-06598-3.

## Introduction

Pluripotent stem cells (PSCs) can self-renew indefinitely and differentiate into cells derived from the three germ layers. While embryonic stem cells (ESCs) are a classical model of PSCs, derived from the inner cell mass of mammalian blastocysts, another type of PSCs, the induced pluripotent stem cells (iPSCs), has arisen in this field in the last decades. These are reprogrammed cells generated from terminally differentiated cells, that constitute a great promise for regenerative medicine. Although there are many clinical trials underway based in cells derived from PSCs [1], there are still many gaps in the knowledge of the molecular mechanisms that govern the pluripotent state and the differentiation processes.

In mouse PSCs, pluripotency is mainly maintained by the Leukemia Inhibitory Factor (LIF) [2], that activates signaling pathways including JAK/STAT3, PI3K/AKT and MEK/ERK [3, 4], which ultimately promote the expression of the core pluripotency transcription factors (TFs) Oct4, Sox2 and Nanog [5, 6].

AKT/PKB kinase plays a central role in the maintenance of different mammalian-derived PSCs [7, 8] and is the main effector of the PI3K/AKT pathway. Although its activation relies on well- characterized events of phosphorylation [9, 10], AKT activity and target specificity are fine-tuned by multiple other post-translational modifications (PTMs) [11–13], including conjugation to the small ubiquitin-related modifier (SUMO) [14–16], referred as SUMOylation, which affects diverse cellular processes [17–20].

SUMOylation is a multiple-step process that involves different specific enzymes, ultimately attaching the SUMO peptide to target lysine residues by the SUMO conjugase enzyme UBC9 [16]. This PTM regulates the function of a plethora of proteins, impacts on protein–protein interactions [21], and can even affect their subcellular localization [22–25]. We have previously reported that SUMOylation of AKT1 is crucial to induce the expression of the pluripotency TF Nanog in mouse ESCs [26]. Additionally, we have recently found that this PTM influences AKT1 subcellular compartmentalization and distribution, and also impacts on NANOG-chromatin binding dynamics in ESCs [27]. As a whole, all these effects dependent on AKT1 SUMOylation could ultimately modulate the gene expression pattern. In this work, we aimed to unravel the underlying molecular mechanism connecting AKT SUMOylation to Nanog gene regulation. For this purpose, we explored the role of both the cellular context and strong candidate TFs that could possibly mediate this regulation, and performed bioinformatic analysis that provided new clues.

## Results and discussion

### AKT1 induces Nanog in a SUMOylation dependent manner in pluripotent contexts

**Fig. 1:**
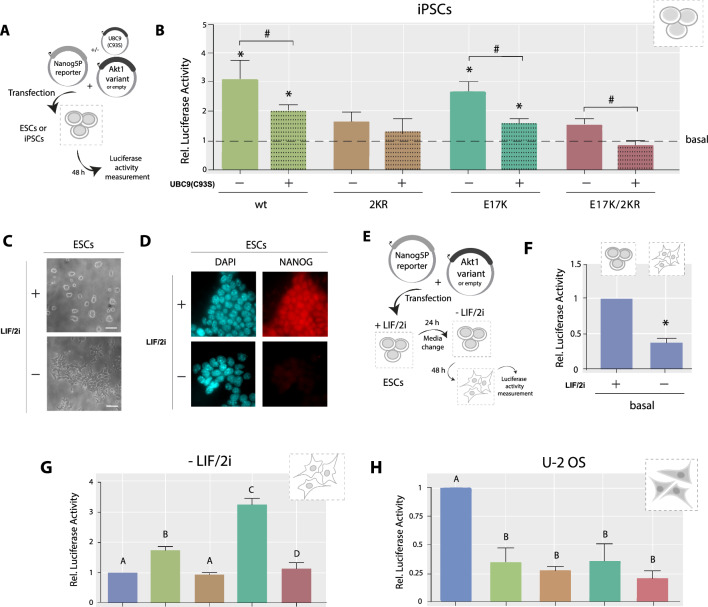
AKT induces Nanog promoter in a SUMOylation-dependent manner in PSCs. **A** Schematic diagram of the experimental design. **B** iPSCs were transfected with either an AKT1 variant (indicated under each bar) or the empty vector (basal), along with the Nanog5P luciferase reporter. A vector encoding the dominant negative UBC9(C93S) was included (+ , dot-patterned bars) or not (−), as indicated. **C**, **D** ESCs were cultured in standard conditions (+ LIF/2i) or were induced to differentiate by LIF/2i withdrawal for 48 h, and then fixed for immunostaining against NANOG. Representative images of phase-contrast microscopy (**C**) and epifluorescence microscopy (**D**). Nuclei were stained with DAPI. **E** Schematic diagram of the experimental design for luciferase experiments at early differentiation. **F**, **G** ESCs were transfected with either an AKT1 variant or the empty vector (basal), along with the Nanog5P luciferase reporter and cultured for 48 h in -LIF/2i medium. **H** U2-Os cells transfected with the vector indicated under each bar, along with the Nanog5P luciferase reporter. In all cases, results were referred to the corresponding basal of each condition (B: dashed line, F: + LIF/2i, G and H: basal) and are shown as mean ± SEM of three independent experiments. When corresponding, different letters indicate significant differences among treatments (p < 0.05), asterisks indicate significant differences of each condition compared to the basal (p < 0.05), and hashes indicate significant differences between fold change of the same AKT1 variant in the experiments comparing with ( +) and without (-) Ubc9(C93S) (p < 0.05). Scale bars: 100 µm

We have previously reported that AKT1 induces Nanog expression in a SUMOylation-dependent manner in mouse ESCs [26]. Interestingly, we found a SUMOylation-independent repression of Nanog promoter by AKT in the terminally differentiated cell line NIH/3T3 of mouse embryonic fibroblasts (MEFs), suggesting that AKT induction of Nanog promoter activity is not a universal effect [26]. These results led us to deepen into the cellular context dependence of this effect, looking for a hint about specific players or the underlying molecular mechanism. We hypothesized that this induction is exclusive of pluripotency contexts, so we reasoned that it could be extended to other pluripotent cells in contrast to differentiated systems. Thus, we first decided to evaluate the effect of AKT1 variants with different SUMOylatability on the Nanog promoter activity in another pluripotent context. As previously mentioned, the iPSCs are reprogrammed PSCs derived from terminally differentiated cells, in many cases from fibroblasts [28], and their gene expression pattern and general properties recall to ESCs. These PSCs express Nanog, and their endogenous promoter is associated with active epigenetic marks, in contrast to the abovementioned MEFs (Additional file 1: Figure S1). As depicted in Fig. 1A, we co-transfected an iPSCs line previously generated in our lab from MEFs [29] with the Nanog5P reporter, a widely used luciferase reporter of the Nanog promoter activity [30], along with the corresponding expression vector encoding a specific AKT1 variant or the empty vector. We used the SUMOylatable AKT1 variants wt and the hyperactive E17K AKT1 mutant, found in various human cancers [31] that exhibits higher SUMOylation levels than the wt [18]; and the corresponding SUMOylation-deficient AKT1 mutants; one derived from the wt AKT1 in which the SUMOylatable lysines 276 (K276) and 301 (K301) were replaced by arginines (2KR AKT1) [18], and other derived from the E17K AKT1 that combines these three mutations (E17K/2KR AKT1). The effects of these AKT1 variants on Nanog expression previously reported in ESCs are summarized in Additional file 1: Figure S2. As shown in Fig. 1B (solid bars) the iPSCs transfected with the SUMOylatable AKT1 variants, wt and E17K AKT1, displayed increased Nanog reporter activity, whereas both SUMOylation-deficient mutants, 2KR and E17K/2KR AKT1 had no effect. Furthermore, when we co-transfected the UBC9(C93S) mutant to interfere with the SUMO-conjugation pathway, the inductor effect of the SUMOylatable variants was impaired (Fig. 1B, dotted bars). Altogether, these results in iPSCs, along with the previously obtained in ESCs [26], demonstrate the SUMOylation-dependence of AKT1 for the induction of the Nanog promoter in different pluripotent contexts.

We reasoned that this induction might be mediated by at least one specific factor present in the pluripotency contexts but absent in differentiated cells. Therefore, we decided to study ESCs at an early differentiation stage. Within the first 48 h of differentiation induction by LIF/2i withdrawal (-LIF/2i), clear morphological changes are triggered (Fig. 1C) and NANOG is almost undetectable (Fig. 1D), as expected [32, 33]. We analyzed the Nanog5P reporter activity after 48 h of differentiation induction (Fig. 1E). As expected, the basal luciferase activity was lower in differentiating cells than in cells cultured in standard conditions (+ LIF/2i) (Fig. 1F). Surprisingly, when evaluating the effect of AKT1 variants, we found the same profile obtained in the naïve pluripotency context, i.e. the AKT1 SUMOylatable variants induced Nanog reporter while the SUMOylation-impaired mutants had no effect (Fig. 1G). This outcome evidenced a conserved mechanism in PSCs even at early stages of differentiation, when Nanog is already downregulated, but Oct4 and Sox2 are still on [32, 33].

Finally, since many tumor-derived cell lines display stemness features [34, 35], we decided to explore the AKT effect on Nanog reporter in a non-pluripotent tumoral context; the osteosarcoma-derived U-2 OS cell line, in which Nanog expression cannot be detected (Additional file 1: Figure S3). The features present in this model provide a different cell context with respect to the other systems studied, thus could provide some clues about the elusive mediator. Interestingly, we found a SUMOylation-independent repression of Nanog promoter (Fig. 1H), evidencing again a different response in this scenario and recalling the observations in MEFs [26].

Overall, our results demonstrate that the SUMOylation-dependent AKT1 induction of Nanog promoter occurs in PSCs, even at early stages of differentiation, but not in non-pluripotent contexts strongly suggesting the requirement of a crucial factor present in PSCs but probably missing in non-pluripotent scenarios.

### The role of the pluripotency TFs OCT4 and SOX2 in the SUMOylation-dependent induction of NANOG by AKT1

**Fig. 2:**
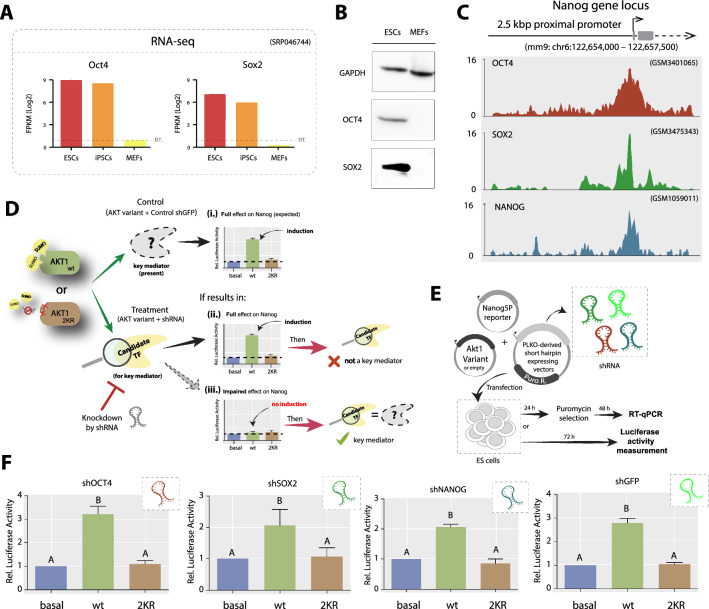
Downregulation of the pluripotency TFs Oct4, Sox2 or Nanog does not interfere with the SUMOylation-dependent AKT induction of Nanog promoter activity. **A** Oct4 and Sox2 gene expression analysis from an RNA-seq experiment in ESCs, iPSCs, and MEFs seq [45]. Data analysis was performed in Stemformatics data-mining platform from publicly available data. DT: detection threshold. **B** Cropped images of western blot analysis of protein extracts of NIH/3T3 MEFs and ESCs, evaluating the presence of OCT4 and SOX2. GAPDH was revealed as loading control (Full blots are shown in Additional file 1: Figure S4). **C** Visualization of the representative enrichment profiles (reads per million) of the indicated TFs in the 2.5 kbp region of the Nanog genomic locus included in Nanog5P reporter. These results correspond to the analysis of publicly available ChIP-seq data from experiments performed in ESCs retrieved from the Chip Atlas database [48–50]. Data was visualized using the Integrative Genomics Viewer (IGV) software. **D** Flowchart representing the rationale of the study to evaluate possible mediators of the SUMOylation-dependent AKT induction of Nanog reporter. Specifically, we evaluated the involvement of the pluripotency TFs through shRNA-mediated knockdown. Panel i shows the control case in which no factor is interfered, thus resulting in the induction of Nanog promoter by wt AKT due to the presence of the active mediator within the cell context. Panel ii shows the case when performing an inhibition of a specific factor not involved in this regulation, obtaining a similar response to the control condition. Panel iii shows that in the case of interfering with a key mediator, no effect on Nanog promoter by wt AKT is expected. Induction of the reporter by 2KR AKT1 is not expected in any of these cases. **E** Schematic diagram of the experimental design. **F** ESCs transfected with either an AKT1 variant or the empty vector (basal), along with the Nanog5P, and the pLKO.1-puro derived vectors targeting either eGFP, Oct4, Sox2 or Nanog, as indicated. The results were referred to the control condition (basal) and are shown as mean ± SEM of three independent experiments. Different letters indicate significant differences (p < 0.01)

The core pluripotency TFs OCT4, SOX2 and NANOG itself are involved in Nanog gene regulation [6, 36–38]. The phosphorylation by different kinases, including AKT, impacts on the activity of these TFs [39–44], so we speculated that they might have a role in the effect observed in PSCs. We verified that these two core TFs are expressed in PSCs but not in MEFs by a comparison analysis from publicly available RNA-seq [45] (Fig. 2A) and by western blot (Fig. 2B, and full blots in Additional file 1: Figure S4). The fact that OCT4 and SOX2 are expressed in naïve PSCs and within the first 48 h of induction of differentiation by LIF/2i withdrawal in ESCs [33, 46], conditions in which we have found the SUMOylation-dependent AKT1 induction of Nanog, and that these TFs are absent in both MEF and U-2 OS cell lines (Additional file 1: Figure S4), where this induction does not occur, designate OCT4 and SOX2 as possible mediators. We decided to explore the involvement of these TFs and included Nanog as a control, since we do not expect this TF to play a role in this induction given that it is greatly downregulated at early differentiation, condition in which we detected the AKT induction on Nanog promoter [33].

We first studied if these TFs effectively bind to the Nanog promoter region included in the Nanog reporter used. We exploited the ChIP Atlas Tool [47] to analyze public data from previous experiments of ChIP-seq performed in ESCs [48–50] and confirmed that OCT4, SOX2 and NANOG bind to the region of the Nanog promoter included in the reporter (Fig. 2C). We hypothesized that the interference of a key mediator of this effect would impede the observed induction; thus, we decided to downregulate these candidate TFs. The scheme in Fig. 2D summarizes the expected outputs for this experimental approach. We downregulated the three pluripotency TFs by transfection of specific shRNA vectors or a control shRNA targeting GFP (shGFP), as depicted in Fig. 2D and E. The efficacy of each shRNA showed a significant reduction in the expression of each of the TFs evaluated compared to the shGFP (Additional file 1: Figure S5), similar to our previous works [51–57]. Surprisingly, none of the shRNA targeting Oct4, Sox2 or Nanog, modified the previously reported response of the Nanog promoter to AKT compared to the control shRNA (Fig. 2F), strongly suggesting that none of these TFS are exclusive mediators, opening a wide range of possible pathways that might be involved in this regulation.

### Screening transcriptomic analysis reveals additional candidate mediators

**Fig. 3:**
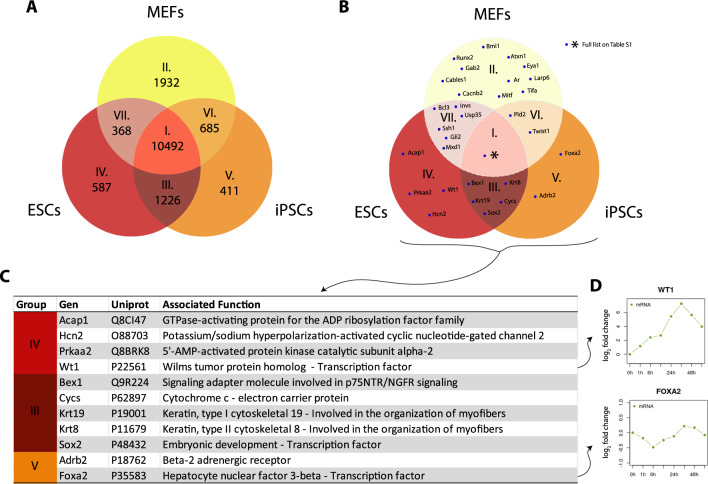
Screening transcriptomic analysis of ESCs, iPSCs and MEFs reveals additional candidate mediators. Analysis of a publicly available dataset of a genome-wide RNA-seq experiment containing paired transcriptomic data of ESCs, iPSCs, and MEFs (SRP046744) [45]. **A** Venn diagram depicting expressed genes above the threshold, showing the number of transcripts, either exclusive or shared, among the three cell types. Groups are arbitrarily numbered from I to VII depending on their size. **B** Exploratory cross-matching analysis between the transcript groups with a to-the-date updated and curated list of AKT1 targets from *PhosphoSitePlus* database. **C** Associated function of the genes from cross-matching expressed in PSCs (ESCs + iPSCs). **D** Foxa2 and Wt1 mRNA expression levels (log_2_ fold change) from an RNA-seq analysis of ESCs differentiating to epiblast-like cells. Data analysis was performed in the Stem Cell Atlas data-mining platform from publicly available multi-omic data [59]

These results, along with our previous findings that ruled out TBX3, GSK3-β and MEK pathways as mediators of the SUMOylation-dependent Nanog induction by AKT1 [26], lead us to consider a new approach to get a hint to unveil the yet elusive molecular mechanism underlying this regulation. Since the inductor effect of SUMOylatable AKT1 on Nanog promoter observed in PSCs is absent in MEFs, we hypothesized that at least a crucial factor shall be present both in ESCs and iPSCs but absent in MEFs. To detect differences that may provide a clue on the sought mediator, we performed a screening by analyzing a publicly available dataset from a genome-wide RNA-seq experiment containing transcriptomic data of MEF, ESCs and iPSCs [45]. Figure 3A shows the expression footprint for each cell type, depicting the specific and shared genes expressed in the different scenarios. We defined groups I to VII, based on the cell type in which they are expressed, and ordered by the number of genes contained. To shorten our list, we then retrieved a to-this-date updated and curated list of AKT1 targets from *PhosphoSitePlus* database [58], and performed an exploratory cross-matching analysis of the different groups (Fig. 3B). We are aware that some AKT1 targets could not have been yet either identified or included in the database. As expected, most of the AKT1 targets were mapped within the group containing the shared transcripts among the three cell types (group I), and just a few located on the rest of the groups. Those AKT1 targets on groups III, IV and V, which are exclusive of pluripotent stem cells, cell contexts in which we detected the SUMOylation-dependent induction, are of our interest and are shown in Fig. 3C. Although, none of them have been yet directly linked to Nanog expression in mouse ESCs, except for Sox2, we believe they constitute an excellent group of candidates for future analysis, especially those factors involved in signaling or identified as TFs, specifically Foxa2 and Wt1. Interestingly, these TFs remain expressed at early stages of a differentiation protocol to epiblast-like cells (EpiLCs) as revealed by analysis of publicly available omics data [59] (Fig. 3D), compatible with the induction of Nanog by AKT1 even at early differentiation stages (Fig. 1G). The expression profile of the remaining candidate genes listed in Fig. 3C is shown in Additional File 1: Figure S6 . Particularly, WT1 is expressed in several kinds of cancers, it is associated with PI3K/AKT pathway and maintenance of stem cell features of cancer stem cells [60], and is also relevant during embryogenesis [61]. On the other hand, Foxa2 is a pioneer TF involved in differentiation [62], and remarkably, its phosphorylation by AKT promotes its nuclear exclusion and inhibition of its transcriptional activity in different contexts [63, 64]. Since Foxa2 was also reported to recruit corepressors [65], we speculate that SUMOylated AKT1 could indirectly induce Nanog expression by promoting the nuclear exclusion of Foxa2 in pluripotent contexts. Further research needs to be performed to elucidate the molecular mechanism underlying this regulation.

## Conclusion

Our results strengthen the importance of the cell context for the AKT1 SUMOylation-dependent regulation of Nanog gene, demonstrating that a crucial factor connected to AKT SUMOylation and Nanog expression is present within the different PSCs contexts and not in the terminally differentiated cell lines studied. Bioinformatics analysis provided new clues for further research.

## Limitations

Even unlikely, we cannot completely discard OCT4 and SOX2 as mediators since we downregulated these TFs but their remaining low levels could be sufficient to mediate the effect. We detected by RT-qPCR around 30% of mRNA of each TF remaining after the knockdown (Additional File 1: Figure S5).

## Methods

Cell culture, transfection, luciferase activity measurement, gene expression analysis and statistical analysis were performed as described previously [26, 27, 33, 54, 66]. Further details and bioinformatic analysis procedures are detailed in Additional File 1: Additional Methods.

### Supplementary Information

**Additional file 1: Figure S1.** Nanog expression and promoter state in MEF and PSCs. Data analysis of Nanog expression and epigenetic marks on its promoter in ESCs, iPSCs and MEFs. Stemformatics data-mining platform was used to perform the analysis from publicly available data [1, 2]. Nanog expression levels from RNA-seq (transcript) and LC-MS (protein) are shown in the upper panel; epigenetic marks within the Nanog promoter region from Histone ChIP-seq (H3K4me3 and H3K27me3, associated to active promoters and repressive marks, respectively) and from cytosine methylation by Bisulfite-sequencing are shown in the lower panel. **Figure S2.** Summary of SUMOylatability and previous results of the AKT variants used. Schematic representations of the AKT1 variants used in our current and previous work and table summarizing their main features (SUMOylation capability [3] and effect on the Nanog promoter in ESCs [4]). SUMO is represented by yellow balloons, hyperactivity-inducing mutations are portrayed as spiny edges and the two lysines replaced by arginine in 2KR and E17K/2KR mutants are indicated by the red Xs. **Figure S3.** NANOG is not detected in the U-2 OS cell line. Representative epifluorescence microscopy images of NANOG immunofluorescence (IF) of the tumoral osteosarcoma U-2 OS cell line, transfected with a vector encoding eGFP-NANOG. Transfection and IF were performed as previously described [4]. Scale bar represents 10 μm. The images show that endogenous NANOG is not detected, since the non-transfected cell (left, yellow arrow), evidenced by DAPI staining, has no NANOG signal (right panel). As a control of the IF, cells were transfected with a vector encoding NANOG fused to the fluorescent protein eGFP (eGFP-NANOG). This fusion protein was detected both through detection of eGFP fluorescence (middle panel) and by IF against NANOG (right panel). **Figure S4.** OCT4 and SOX2 are not detected in MEFs and U-2 OS cells. Western blot analysis of protein extracts of ESCs, NIH/3T3 MEFs and U-2 OS evaluating the presence of OCT4 and SOX2. GAPDH was revealed as loading control. The sample loaded in the 3rd lane corresponds to a cell line that is not included in this work. **Figure S5.** Downregulation of Oct4, Sox2 and Nanog by shRNA. ESCs were transfected with the pLKO.1-puro derived vectors encoding shRNA targeting Oct4 (shOct4), Sox2 (shSox2), Nanog (shNanog) and eGFP (shGFP), as indicated below each bar. mRNA levels of each specific target, indicated at the top, were evaluated by RT-qPCR, normalized to the geometric mean of Gapdh and Pgk1 and referred to the control (shGFP). Bars represent the mean ± SEM of three independent experiments. Asterisks (*) indicate significant differences compared to the corresponding control condition (p < 0.05). **Figure S6.** Multi-omic analysis of genes from Groups III to V in ESCs differentiating to epiblast-like cells (Related to Figure 3). mRNA (green) and/or protein (light blue) expression levels (log2 fold change) from multi-omic analysis of ESCs differentiating to epiblast-like cells. Data analysis was performed in the Stem Cell Atlas data-mining platform (http://www.stemcellatlas.org/) from publicly available transcriptomic and proteomic data [5]. **Table S1.** Full gene list of Group I from the cross-matching analysis of Figure 3.

## Data Availability

The data that support the findings of this study are available from the corresponding author upon reasonable request.

## Abbreviations

ESCs: Embryonic stem cells
iPSCs: Induced pluripotent stem cells
PSCs: Pluripotent stem cells
MEF: Mouse embryonic fibroblast
LIF: Leukemia Inhibitory Factor
PTM: Post-translational modification
shRNA: Short hairpin RNA
SUMO: Small ubiquitin-related modifier
TF: Transcription factor
