# Supplementary material for: AKT1 induces Nanog promoter in a SUMOylation-dependent manner in different pluripotent contexts
Source: BMC Res Notes. 2023 Nov 3;16:309. doi: 10.1186/s13104-023-06598-3 (PMC10623886; doi:10.1186/s13104-023-06598-3)
Supplement: Supplementary file 1 — Additional file 1: Figure S1. Nanog expression and promoter state in MEF and PSCs. Data analysis of Nanog expression and epigenetic marks on its promoter in ESCs, iPSCs and MEFs. Stemformatics data-mining platform was used to perform the analysis from publicly available data [1, 2]. Nanog expression levels from RNA-seq (transcript) and LC-MS (protein) are shown in the upper panel; epigenetic marks within the Nanog promoter region from Histone ChIP-seq (H3K4me3 and H3K27me3, associated to active promoters and repressive marks, respectively) and from cytosine methylation by Bisulfite-sequencing are shown in the lower panel. Figure S2. Summary of SUMOylatability and previous results of the AKT variants used. Schematic representations of the AKT1 variants used in our current and previous work and table summarizing their main features (SUMOylation capability [3] and effect on the Nanog promoter in ESCs [4]). SUMO is represented by yellow balloons, hyperactivity-inducing mutations are portrayed as spiny edges and the two lysines replaced by arginine in 2KR and E17K/2KR mutants are indicated by the red Xs. Figure S3. NANOG is not detected in the U-2 OS cell line. Representative epifluorescence microscopy images of NANOG immunofluorescence (IF) of the tumoral osteosarcoma U-2 OS cell line, transfected with a vector encoding eGFP-NANOG. Transfection and IF were performed as previously described [4]. Scale bar represents 10 μm. The images show that endogenous NANOG is not detected, since the non-transfected cell (left, yellow arrow), evidenced by DAPI staining, has no NANOG signal (right panel). As a control of the IF, cells were transfected with a vector encoding NANOG fused to the fluorescent protein eGFP (eGFP-NANOG). This fusion protein was detected both through detection of eGFP fluorescence (middle panel) and by IF against NANOG (right panel). Figure S4. OCT4 and SOX2 are not detected in MEFs and U-2 OS cells. Western blot analysis of protein extracts of ESCs, NIH/3T3 M [file 13104_2023_6598_MOESM1_ESM.pdf]

Additional File 1

AKT1 induces Nanog promoter in a SUMOylation-dependent manner in different pluripotent contexts

Marcos Gabriel Francia, Paula Verneri, Camila Oses, Camila Vazquez Echegaray, Mora Reneé Garcia, Ayelen Toro, Valeria Levi, Alejandra Sonia Guberman.

1. Additional Figures

2. Additional Table

3. Additional Methods

4. Additional References

1. Additional Figures

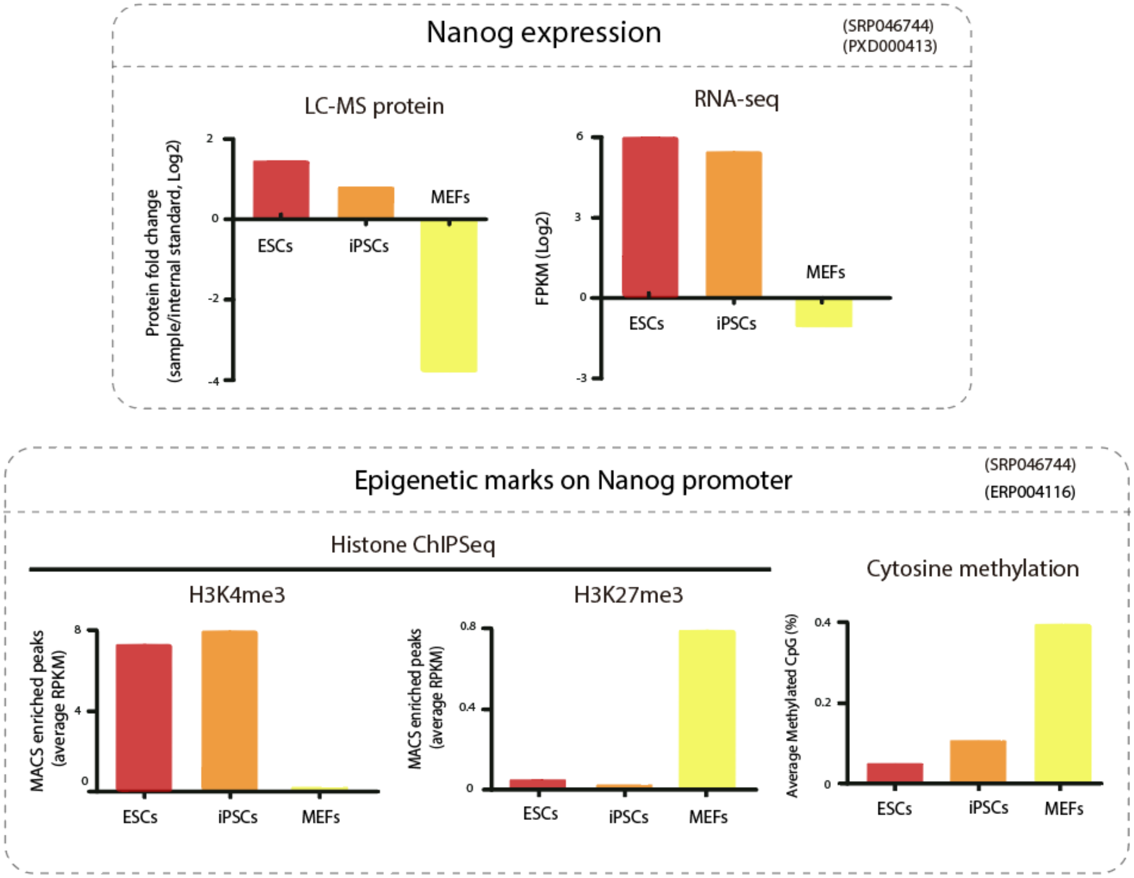

Figure S1. Nanog expression and promoter state in MEF and PSCs. Data analysis of Nanog expression and epigenetic marks on its promoter in ESCs, iPSCs and MEFs. Stemformatics data-mining platform was used to perform the analysis from publicly available data [1, 2]. Nanog expression levels from RNA-seq (transcript) and LC-MS (protein) are shown in the upper panel; epigenetic marks within the Nanog promoter region from Histone ChIP-seq (H3K4me3 and H3K27me3, associated to active promoters and repressive marks, respectively) and from cytosine methylation by Bisulfite-sequencing are shown in the lower panel.

AKT1 variant

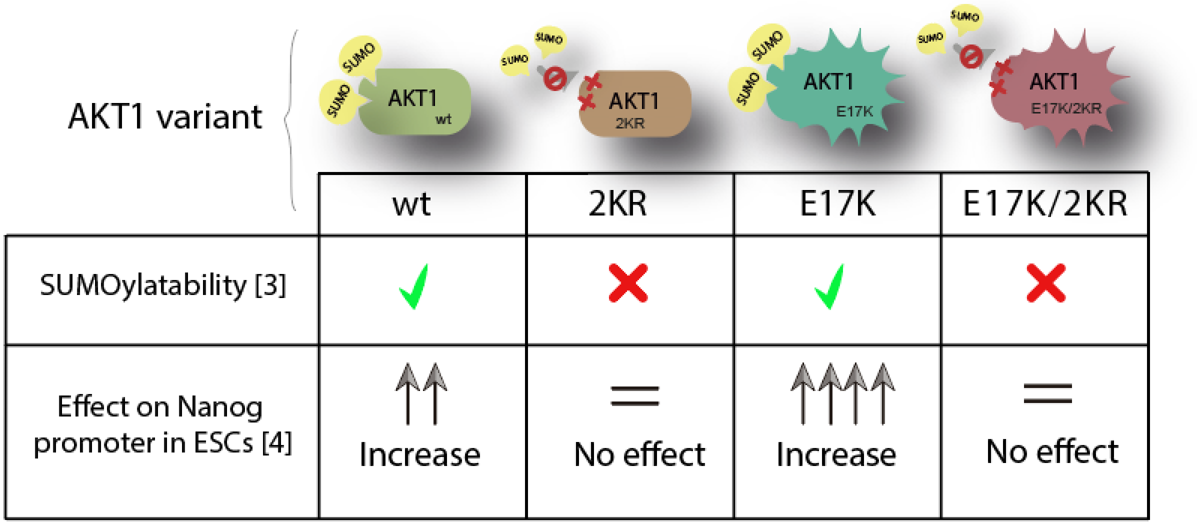

	wt	2KR	E17K	E17K/2KR
SUMOylatability [3]	✓	✗	✓	✗
Effect on Nanog promoter in ESCs [4]	↑↑ Increase	= No effect	↑↑↑↑ Increase	= No effect

Figure S2. Summary of SUMOylatability and previous results of the AKT variants used. Schematic representations of the AKT1 variants used in our current and previous work and table summarizing their main features (SUMOylation capability [3] and effect on the Nanog promoter in ESCs [4]). SUMO is represented by yellow balloons, hyperactivity-inducing mutations are portrayed as spiny edges and the two lysines replaced by arginine in 2KR and E17K/2KR mutants are indicated by the red Xs.

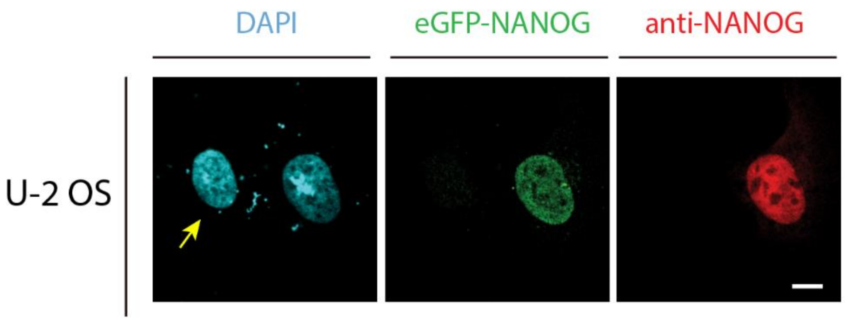

Figure S3. NANOG is not detected in the U-2 OS cell line. Representative epifluorescence microscopy images of NANOG immunofluorescence (IF) of the tumoral osteosarcoma U-2 OS cell line, transfected with a vector encoding eGFP-NANOG. Transfection and IF were performed as previously described [4]. Scale bar represents 10 µm. The images show that endogenous NANOG is not detected, since the non-transfected cell (left, yellow arrow), evidenced by DAPI staining, has no NANOG signal (right panel). As a control of the IF, cells were transfected with a vector encoding NANOG fused to the fluorescent protein eGFP (eGFP-NANOG). This fusion protein was detected both through detection of eGFP fluorescence (middle panel) and by IF against NANOG (right panel).

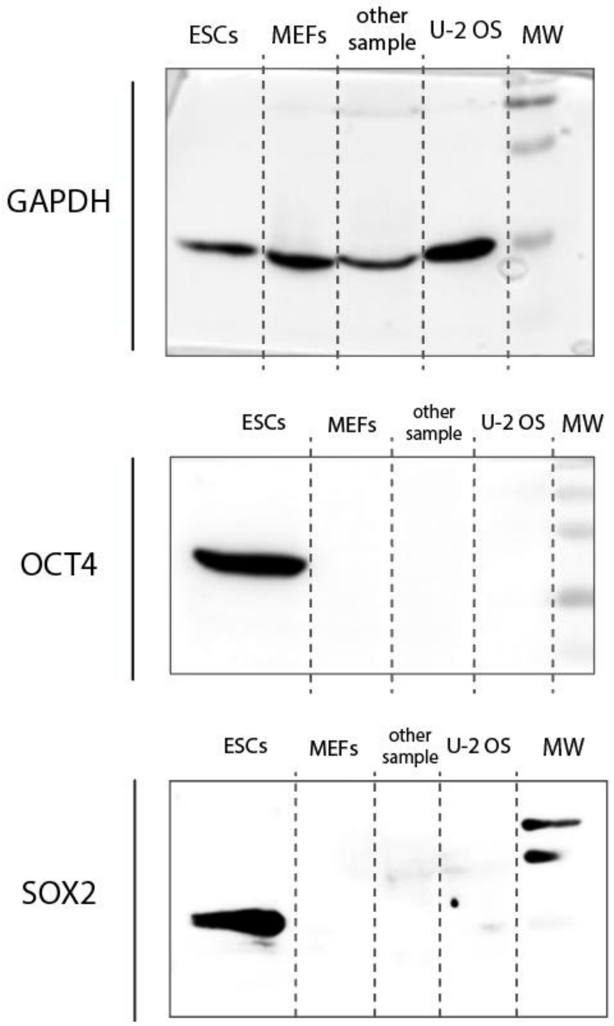

Figure S4. OCT4 and SOX2 are not detected in MEFs and U-2 OS cells. Western blot analysis of protein extracts of ESCs, NIH/3T3 MEFs and U-2 OS evaluating the presence of OCT4 and SOX2. GAPDH was revealed as loading control. The sample loaded in the 3rd lane corresponds to a cell line that is not included in this work.

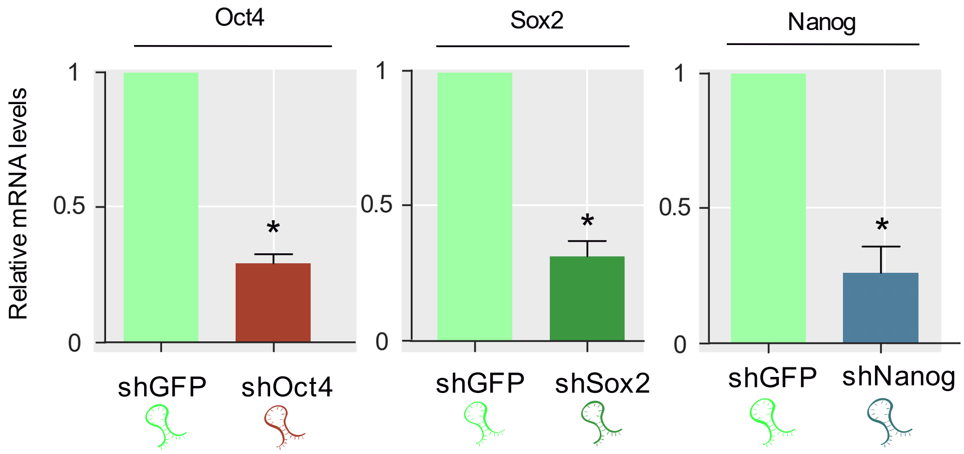

Figure S5. Downregulation of Oct4, Sox2 and Nanog by shRNA. ESCs were transfected with the pLKO.1-puro derived vectors encoding shRNA targeting Oct4 (shOct4), Sox2 (shSox2), Nanog (shNanog) and eGFP (shGFP), as indicated below each bar. mRNA levels of each specific target, indicated at the top, were evaluated by RT-qPCR, normalized to the geometric mean of Gapdh and Pgk1 and referred to the control (shGFP). Bars represent the mean ± SEM of three independent experiments. Asterisks (*) indicate significant differences compared to the corresponding control condition ($p < 0.05$).

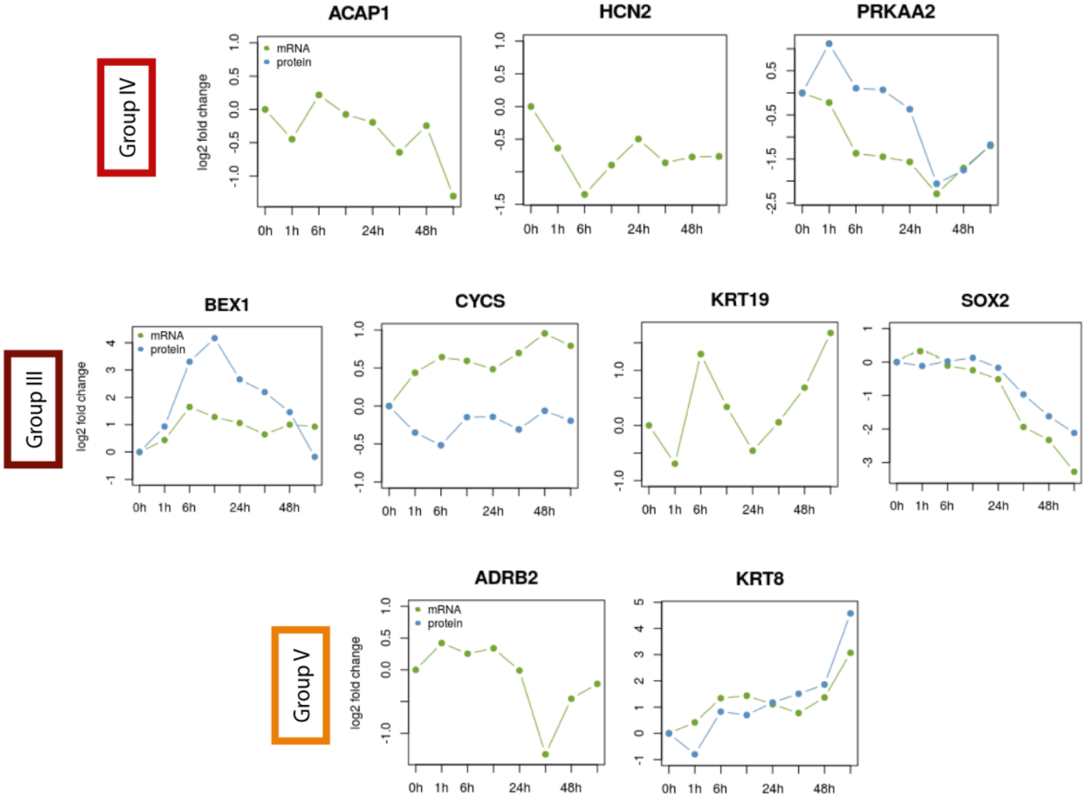

Figure S6. Multi-omic analysis of genes from Groups III to V in ESCs differentiating to epiblast-like cells (Related to Figure 3). mRNA (green) and/or protein (light blue) expression levels (log2 fold change) from multi-omic analysis of ESCs differentiating to epiblast-like cells. Data analysis was performed in the Stem Cell Atlas data-mining platform (http://www.stemcellatlas.org/) from publicly available transcriptomic and proteomic data [5].

2. Additional Table

Acin1	Cdca7	Dtnbp1	Hspb1	Mtor	Phf20	Scyl1	Tsc2
Acly	Cdk2	Edc3	Htra2	Ncl	Pikfyve	Sik2	Ttc3
Adar	Celf1	Egfr	Htt	Ncor1	Pkmyt1	Sirt6	Txnip
Adarb1	Cflar	Ep300	Ilf3	Ndrg2	Plcg1	Ski	Ulk1
Akt1	Chek1	Epha2	Irak1	Nf2	Ppat	Skp2	Usp14
Alyref	Chuk	Ezh2	Irs1	Nhej1	Prkaa1	Smad3	Usp4
Arfip2	Clk2	Faf1	Irs2	Npm1	Prpf19	Sp1	Usp8
Arhgap19	Clock	Fbxw7	Itch	Nqo1	Prph	Srpk2	Vcp
Arhgap22	Cops6	Flii	Itgb3	Nuak1	Ptk2	Srsf3	Vdac2
Aurkb	Creb1	Flna	Itpr1	Pacs2	Ptpn1	Ssb	Vim
Bad	Crebbp	Flnc	Iws1	Pak1	Ptpn6	Stk3	Wee1
Bax	Cs	Foxo1	Khsrp	Palld	Pygo2	Stk4	Wnk1
Bcl10	Csk	Foxo3	Lbr	Park7	Rac1	Stx7	Xiap
Becn1	Ctnnb1	Foxo4	Lgmn	Pawr	Raf1	Tarbp2	Yap1
Braf	Cyth2	Gja1	Lmna	Pcbp1	Ralgapa2	Tcf3	Ybx1
Brca1	Cyth3	Golga3	Lonp1	Pcgf2	Ranbp3	Terf1	Ywhaz
Carhsp1	Depdc5	Grb10	Mapkap1	Pdcd4	Rara	Tert	Zranb1
Casp9	Diablo	Hjurp	Mastl	Pdk1	Rarg	Tfe3	Zyx
Cbx3	Dlc1	Hk2	Maz	Pfkfb2	Rictor	Tfeb	
Cby1	Dnajc5	Hmox1	Mdm2	Pfkfb3	Rnf11	Tiam1	
Ccnf	Dnmt1	Hnrnpa1	Mdm4	Phb	Rps3	Tkt	
Cct2	Dock6	Hsf1	Mettl1	Phb2	Rps6	Topbp1	

Table S1. Full gene list of Group I from the cross-matching analysis of Figure 3.

3. Additional Methods

Cell culture

The W4 mouse ESCs line was supplied by the Rockefeller University Core Facility (NY, USA) and the iPSCs line was previously generated and validated by our group [6]. ESCs and iPSCs were routinely cultured in 0.1 % gelatin-coated dishes with ESC medium containing DMEM, 100 mM MEM NEAA, 2 mM Glutamax, 100 mg/ml streptomycin, 100 U/ml penicillin, 0.1 mM 2-mercaptoethanol, 15% fetal bovine serum (FBS, Gibco), LIF and the two-inhibitors (2i) cocktail consisting of 3 µM CHIR 99021 (Tocris) and 1 µM PD0325901 (Tocris). U-2 OS (ATCC HTB-96) were cultured in DMEM supplemented with 10% FBS (Internegocios), 100 mg/ml streptomycin and 100 U/ml penicillin (Gibco). All the cell lines were maintained at 37 °C in a 5 % CO_2 (v/v) incubator and passed every two or three days. All experiments were performed in these conditions unless otherwise indicated. Mycoplasma contamination was regularly assessed in all cell lines by genomic DNA extraction followed by PCR analysis [7].

Transfection and luciferase activity assay

For the luciferase activity assay, cells were harvested and plated in a 24-well plate with the corresponding medium. ESCs and iPSCs were plated at a density of 36000 cells/well, and U-2 OS at 16000 cells/well. After 24 h, the culture medium was replaced according to the specified conditions of each experiment and cells were transfected with both 650 ng of either the empty vector or the corresponding AKT1 variant encoding vector [3] and 600 ng of the Nanog5P reporter. This luciferase reporter was kindly provided by Austin Cooney (Addgene plasmid # 16337) [8]. PEI (Linear Polyethylenimine 25 kDa, Polysciences Inc.) was used for cell transfection with a DNA/PEI ratio of 1:3, in the case of ESCs and iPSCs, and 1:5 for U-2 OS and NIH/3T3 MEF cells. After overnight incubation, medium was replaced and cells were lysed and assayed for luciferase activity 24 or 48 h later (as indicated), using the Dual Luciferase kit (Promega) on a GloMax Multi Detection System (Promega). Total protein mass was measured by Bradford method and

used for normalization in each transfection assay. Experiments were performed in triplicate and repeated at least three times. No substantial differences were detected in the proportion of transfected cells and the expression levels for the different AKT variants verified by immunofluorescence and Western blot [4]. The downregulation of Nanog, Oct4and Sox2 by short hairpin RNA (shRNA) approach was performed as previously described [9–15]. Briefly, ESCs were transfected and processed as described above, using 500 ng of the corresponding AKT1 variant or the empty vector, 450 ng of Nanog5P reporter, and 600 ng of the pLKO.1-puro derived vectors (Sigma) expressing shRNA targeting Nanog (SHCLND-XM_132755), Oct4 (SHCLND-NM_013633), Sox2 (SHCLND-NM_003106) and eGFP (SHC005), which was used as control shRNA.

Gene expression analysis

Western blot and Immunofluorescence

Protein expression levels were analyzed either by Western blot *(WB) or* immunofluorescence (IF) as previously described [4]. The following antibodies and dilutions were used in this work: Anti-GAPDH for WB (SCBT, SC-32233), Anti-NANOG 1:100 for IF (Peprotech, cat: 500-P236), Anti-OCT4 1:500 for WB and 1:100 for IF (SCBT, SC-5279), Anti-SOX2 1:500 for WB and 1:100 for IF (SCBT, SC-17320), Anti-Rabbit-HRP 1:1000 for WB (SCBT, SC-2357), Anti-Mouse-HRP 1:1000 for WB (thermo), Anti-Goat-HRP for WB 1:1000 (SCBT, SC-2020), Anti-Rabbit-Alexa555 1:1000 for IF (Life technologies, A31572).

RT-qPCR

mRNA expression levels were analyzed by RT-qPCR as previously described [12, 16]. The following primers, targeting mouse transcripts were used in this work: Gapdh: forward primer (FP), 5’ TGCCAAGGCTGTGGGCAAGG ‘3; reverse primer (RP), 5’ CGAAGGTGGAAGAGTGGG ‘3. Nanog: FP, 5’ AGGGTCTGCTACTGAGATGCTCTG ‘3; RP, 5’ CAACCACTGGTTTTTCTGCCACCG ‘3. Oct4: FP, 5’

TGACGGGAACAGAGGGAAAG ‘3; RP, 5’ TCAGCTTGGGCTAGAGAAGG ‘3. Pgk1: FP, 5’ TGGGCAAGGATGTTCTGTTC ‘3; RP, 5’ TGCAGTCCCAAAAGCATCAT ‘3. Sox2: FP, 5’ CACAACTCGGAGATCAGCAA ‘3; RP, 5’ CTCCGGGAAGCGTGTACTTA ‘3.

Bioinformatic Analysis

Expression of pluripotency TFs (Figure S1 and Figure 2A) and epigenetic marks on Nanog promoter (Figure S1) in ESCs, iPSCs and MEF were evaluated using the *Stemformatics* web tool (https://www.stemformatics.org) [17, 18] and exploiting genome-wide publicly available datasets from RNA-seq (SRP046744), LC-MS (PXD000413), H3K4me3 (SRP046744) and H3K27me3 (SRP046744) ChIP-seq, and whole genome bisulfite sequencing (ERP004116) experiments [1, 2]. Methods for data annotation, transformation and normalization are available at Stemformatics documentation (https://www.stemformatics.org/about).

For the analysis of Chromatin Immunoprecipitation (ChIP) assays (Figure 2C), the *ChIP-Atlas* data-mining platform (http://chip-atlas.org) [19] was used. Evaluation of the enrichment profile of OCT4, SOX2 and NANOG in the 2.5 kbp region of the Nanog genomic locus included in Nanog5P reporter was assessed using publicly available ChIP followed by high-throughput sequencing (ChIP-seq) data of experiments performed in ESCs (OCT4: GSM3401065, SOX2: GSM3475343, NANOG: GSM1059011) [20–22]. The Integrative Genomics Viewer (IGV) software [23] was used for data visualization. Data normalization, transformation and annotation methods are available at ChIP-Atlas documentation (https://github.com/inutano/chip-atlas/wiki).

For the analysis in Figure 3, a publicly available dataset of a genome-wide RNA-seq experiment containing paired transcriptomic data of ESCs, iPSCs, and MEFs was retrieved from the *Stemformatics* database (SRP046744) [1]. Annotation, transformation, and normalization of this dataset were previously performed, and procedures are available at Stemformatics documentation

(https://www.stemformatics.org/about) [17, 18]. All transcripts with FPKM above two times the detection threshold were retrieved from the ESCs, iPSCs, and MEFs groups. A comparison of all expressed genes, either shared or non-shared between the groups, were performed for the Venn diagram. Groups were numbered from I to VII depending on the number of genes contained. Then, a to-the-date updated AKT1 targets from the *PhosphoSitePlus* database were retrieved [24]. Procedures of validation and data curation of these targets are available at *PhosphoSitePlus* documentation (https://www.phosphosite.org/staticCurationProcess.action). Subsequently, a cross-matching analysis between the list of AKT1 targets and the transcript groups identified was performed. Details and associated function of each gene were retrieved from the *Uniprot* database [25].

For Figure 3D and Figure S5, data analysis was performed in the Stem Cell Atlas data-mining platform from publicly available transcriptomic and proteomic data [5]. Annotation, transformation, and normalization of this dataset were previously performed, and procedures are available at *Stem Cell Atlas* documentation (http://www.stemcellatlas.org/).

Statistical Analysis

Data was analyzed as previously described [4, 26–28]. Significance between groups was analyzed by linear mixed models (LMM). Residuals fitted normal distribution and homogeneity of variance. Otherwise, transformation of data (log) was applied in some cases to meet both assumptions. Post-hoc multiple comparisons between means were assessed using the Tukey's HSD test. Experimental results were expressed as mean ± standard error of the mean (SEM) of at least three biological replicates. Differences were regarded as significant at least with a p-value of ≤0.05. The statistical analysis was performed using Infostat Software [29] or the gls package of RStudio. Specific analysis information is presented in each figure legend.

4. Additional References

1. Hussein SMI, Puri MC, Tonge PD, Benevento M, Corso AJ, Clancy JL, et al. Genome-wide characterization of the routes to pluripotency. Nature 2014 516:7530. 2014;516:198–206.
2. Benevento M, Tonge PD, Puri MC, Hussein SMI, Cloonan N, Wood DL, et al. Proteome adaptation in cell reprogramming proceeds via distinct transcriptional networks. Nature Communications 2014 5:1. 2014;5:1–11.
3. Risso G, Pelisch F, Pozzi B, Mammi P, Blaustein M, Colman-Lerner A, et al. Modification of Akt by SUMO conjugation regulates alternative splicing and cell cycle. Cell Cycle. 2013;12:3165–74.
4. Francia M, Stortz M, Echegaray CV, Oses C, Verneri P, Petrone MV, et al. SUMO conjugation susceptibility of Akt/protein kinase B affects the expression of the pluripotency transcription factor Nanog in embryonic stem cells. PLoS One. 2021;16:e0254447.
5. Yang P, Humphrey SJ, Cinghu S, Pathania R, Oldfield AJ, Kumar D, et al. Multi-Omic Profiling Reveals Dynamics of the Phased Progression of Pluripotency. Cell Syst. 2019;8:427.
6. Solari C, Losino N, Luzzani C, Waisman A, Bluguermann C, Questa M, et al. Biochemical and Biophysical Research Communications Induced pluripotent stem cells ' self-renewal and pluripotency is maintained by a bovine granulosa cell line-conditioned medium. Biochem Biophys Res Commun. 2011;410:252–7.
7. Uphoff CC, Drexler HG. Detecting Mycoplasma Contamination in Cell Cultures by Polymerase Chain Reaction. Cancer Cell Culture. 2011;731:93–103.
8. Gu P, LeMenuet D, Chung AC-K, Mancini M, Wheeler DA, Cooney AJ. Orphan Nuclear Receptor GCNF Is Required for the Repression of Pluripotency Genes during Retinoic Acid-Induced Embryonic Stem Cell Differentiation. Mol Cell Biol. 2005;25:8507–19.
9. Villodre ES, Felipe KB, Oyama MZ, Oliveira FH de, Lopez PL da C, Solari C, et al. Silencing of the transcription factors Oct4, Sox2, Klf4, c-Myc or Nanog has different effect on teratoma growth. Biochem Biophys Res Commun. 2019;517:324–9.
10. Solari C, Echegaray CV, Luzzani C, Cosentino MS, Waisman A, Petrone MV, et al. Protein arginine Methyltransferase 8 gene is expressed in pluripotent stem cells and its expression is modulated by the transcription factor Sox2. Biochem Biophys Res Commun. 2016;473.
11. Solari C, Petrone MV, Vazquez Echegaray C, Cosentino MS, Waisman A, Francia M, et al. Superoxide dismutase 1 expression is modulated by the core pluripotency transcription factors Oct4, Sox2 and Nanog in embryonic stem cells. Mech Dev. 2018;154:116–21.
12. Cosentino MS, Oses C, Vázquez Echegaray C, Solari C, Waisman A, Álvarez Y, et al. Kat6b Modulates Oct4 and Nanog Binding to Chromatin in Embryonic Stem Cells and Is Required for Efficient Neural Differentiation. J Mol Biol. 2019;431:1148–59.
13. Solari C, Petrone MV, Toro A, Vazquez Echegaray C, Cosentino MS, Waisman A, et al. The pluripotency transcription factor Nanog represses glutathione reductase gene expression in mouse embryonic stem cells. BMC Res Notes. 2019;12:1–7.
14. Petrone MV, Toro A, Vazquez Echegaray C, Francia MG, Solari C, Cosentino MS, et al. The pluripotency transcription factor OCT4 represses heme oxygenase-1 gene expression. FEBS Lett. 2021;595:1949–61.

15. Solari C, Vázquez Echegaray C, Cosentino MS, Petrone MV, Waisman A, Luzzani C, et al. Manganese Superoxide Dismutase Gene Expression Is Induced by Nanog and Oct4, Essential Pluripotent Stem Cells' Transcription Factors. PLoS One. 2015;10:e0144336.
16. Waisman A, Vazquez Echegaray C, Solari C, Cosentino MS, Martyn I, Deglincerti A, et al. Inhibition of Cell Division and DNA Replication Impair Mouse-Naïve Pluripotency Exit. J Mol Biol. 2017;429:2802–15.
17. Wells CA, Mosbergen R, Korn O, Choi J, Seidenman N, Matigian NA, et al. Stemformatics: Visualisation and sharing of stem cell gene expression. Stem Cell Res. 2013;10:387–95.
18. Choi J, Pacheco CM, Mosbergen R, Korn O, Chen T, Nagpal I, et al. Stemformatics: Visualize and download curated stem cell data. Nucleic Acids Res. 2019;47:D841–6.
19. Oki S, Ohta T, Shioi G, Hatanaka H, Ogasawara O, Okuda Y, et al. Ch IP -Atlas: a data-mining suite powered by full integration of public Ch IP -seq data . EMBO Rep. 2018;19.
20. Das PP, Shao Z, Beyaz S, Apostolou E, Pinello L, Angeles ADL, et al. Distinct and combinatorial functions of Jmjd2b/Kdm4b and Jmjd2c/Kdm4c in mouse embryonic stem cells identity. Mol Cell. 2014;53:32.
21. Festuccia N, Owens N, Papadopoulou T, Gonzalez I, Tachtsidi A, Vandoermel-Pournin S, et al. Transcription factor activity and nucleosome organization in mitosis. Genome Res. 2019;29:250–60.
22. Boija A, Klein IA, Sabari BR, Dall'Agnese A, Coffey EL, Zamudio A v., et al. Transcription Factors Activate Genes through the Phase-Separation Capacity of Their Activation Domains. Cell. 2018;175:1842-1855.e16.
23. Robinson JT, Thorvaldsdóttir H, Winckler W, Guttman M, Lander ES, Getz G, et al. Integrative genomics viewer. Nature Biotechnology. 2011;29:24–6.
24. Hornbeck P v., Zhang B, Murray B, Kornhauser JM, Latham V, Skrzypek E. PhosphoSitePlus, 2014: mutations, PTMs and recalibrations. Nucleic Acids Res. 2015;43:D512–20.
25. Bateman A, Martin MJ, Orchard S, Magrane M, Agivetova R, Ahmad S, et al. UniProt: the universal protein knowledgebase in 2021. Nucleic Acids Res. 2021;49:D480–9.
26. Cosentino MS, Oses C, Vázquez Echegaray C, Solari C, Waisman A, Álvarez Y, et al. Kat6b Modulates Oct4 and Nanog Binding to Chromatin in Embryonic Stem Cells and Is Required for Efficient Neural Differentiation. J Mol Biol. 2019;431:1148–59.
27. Verneri P, Vazquez Echegaray C, Oses C, Stortz M, Guberman A, Levi V, et al. Dynamical reorganization of the pluripotency transcription factors Oct4 and Sox2 during early differentiation of embryonic stem cells. Sci Rep. 2020;10:1–12.
28. Toro A, Anselmino N, Solari C, Francia M, Oses C, Sanchis P, et al. Novel Interplay between p53 and HO-1 in Embryonic Stem Cells. Cells 2021, Vol 10, Page 35. 2020;10:35.
29. JA di Rienzo, F Casanoves, Balzarini M, Gonzalez L, Tablada M, Robledo C. Infostat - Sofware estadístico. Universidad Nacional de Córdoba, Argentina. Universidad Nacional de Córdoba, Argentina. 2013.
